# Institutional strategies related to test-taking behavior in low stakes assessment

**DOI:** 10.1007/s10459-019-09928-y

**Published:** 2019-10-22

**Authors:** Katrin Schüttpelz-Brauns, Martin Hecht, Katinka Hardt, Yassin Karay, Michaela Zupanic, Juliane E. Kämmer

**Affiliations:** 1 Medical Faculty Mannheim, Heidelberg University, Theodor-Kutzer-Ufer 1-3, 68167 Mannheim, Germany; 2 Institute of Cognitive and Clinical Neuroscience, Central Institute for Mental Health in Mannheim, J5, 68159 Mannheim, Germany; 3 Faculty of Life Sciences, Humboldt-Universität zu Berlin, Unter den Linden 6, 10099 Berlin, Germany; 4 Medical Faculty, University of Cologne, Joseph-Stelzmann-Straße 20 (Building 42), 50931 Cologne, Germany; 5 Faculty of Health, Witten/Herdecke University, Alfred-Herrhausen-Straße 50, 58448 Witten, Germany; 6 AG Progress Test Medizin, Charité Universitätsmedizin Berlin, Hannoversche Straße 19, 10115 Berlin, Germany; 7 Center for Adaptive Rationality, Max Planck Institute for Human Development, Lentzeallee 94, 14195 Berlin, Germany

**Keywords:** Curriculum development, Low stakes assessment, Progress testing, Self-determination theory, Test-taking effort

## Abstract

Low stakes assessment without grading the performance of students in educational systems has received increasing attention in recent years. It is used in formative assessments to guide the learning process as well as in large-scales assessments to monitor educational programs. Yet, such assessments suffer from high variation in students’ test-taking effort. We aimed to identify institutional strategies related to serious test-taking behavior in low stakes assessment to provide medical schools with practical recommendations on how test-taking effort might be increased. First, we identified strategies that were already used by medical schools to increase the serious test-taking behavior on the low stakes Berlin Progress Test (BPT). Strategies which could be assigned to self-determination theory of Ryan and Deci were chosen for analysis. We conducted the study at nine medical schools in Germany and Austria with a total of 108,140 observations in an established low stakes assessment. A generalized linear-mixed effects model was used to assess the association between institutional strategies and the odds that students will take the BPT seriously. Overall, two institutional strategies were found to be positively related to more serious test-taking behavior: discussing low test performance with the mentor and consequences for not participating. Giving choice was negatively related to more serious test-taking behavior. At medical schools that presented the BPT as evaluation, this effect was larger in comparison to medical schools that presented the BPT as assessment.

**Electronic supplementary material:**

The online version of this article (10.1007/s10459-019-09928-y) contains supplementary material, which is available to authorized users.

## Introduction

Low stakes assessment without grading the performance of students in educational systems has received increasing attention in recent years. Such assessments serve two purposes: first, they are meant to guide the learning process as a formative assessment (assessment for learning), which can increase learning effects (Black and William 1998; Hattie and Timperley 2007; Kluger and DeNisi 1996; Shute 2008), and they are an important part of self-regulated learning (Ecclestone 2010; Irons 2008; Nicol and Macfarlance-Dick^1999^; White and Gruppen 2010). Second, low stakes tests are used in large-scale assessment programs that are part of the quality management of educational monitoring (Campbell et al. 1998; Organization for Economic Cooperation and Development 1999, 2003) and thus have a substantial impact on educational reform, policy making, and resource allocation (Breakspear 2012; Fullan 2009). Low stakes assessment, however, suffers from high variation in test-taking effort, as students may not be willing to give their best effort to take the test (Wise and DeMars 2010), resulting in less serious test-taking behavior. As a consequence of this high variation, low stakes assessment may not actually serve these two purposes adequately.

One important example for low stakes assessment are progress tests in medicine that are used as both large-scale (Edwards et al. 2014; Muijtjens et al. 2008; Nouns et al. 2012) and formative assessment (Freeman et al. 2010; Muijtjens et al. 2010; Nouns and Georg 2010) by many institutions, such as the German and Austrian consortium of 16 medical schools (Freeman et al. 2010; Nouns and Georg 2010; Osterberg et al. 2006; Wrigley et al. 2012). Progress tests in medicine are tests that assess the knowledge that a recently graduated physician needs on his/her first day. These progress tests are administered repeatedly during undergraduate training. Students take the same test for all levels of the undergraduate medical training (e.g., Blake et al. 1996; Freeman et al. 2010; Nouns and Georg 2010; van Berkel et al. 1993). Participating students can see their progress in knowledge over the course of their undergraduate training in comparison to their peers who have completed the same curriculum. Thus, they can identify strengths and deficits and consequently are able to focus on their learning activities. Faculties can use the information to evaluate, develop, and compare their curricula (Freeman et al. 2010; Nouns et al. 2012; van der Vleuten et al. 2004).

The German and Austrian progress test consortium (Nouns and Georg 2010) provides a progress test (Berlin Progress Test, BPT) where individual performance does not have consequences for the students. The proportion of students who take the test seriously is routinely analyzed as an indicator of test-taking effort and shows a wide range of results among the participating medical schools (range 44–100%).

For years, medical faculties have been trying to increase serious test-taking behavior by (more or less theory-based) implementing different strategies such as punishing non-participation, discussing a low test performance with the mentor or stressing the value of reliable, continuous feedback. Most of these strategies can be post hoc classified into a theoretical framework that can explain motivational behavior in educational settings (Kusurkar et al. 2011), which is closely connected to test-taking effort. Kusurkar et al. (2011) summarized motivational theories that were useful in educational contexts and found that most of them focus on the level of motivation, but not on the quality of motivation. They concluded that only Ryan and Deci’s (2000) self-determination theory (SDT) “is a general motivation theory which holds true for different aspects of motivation in an individual’s life, including education and learning” (p. 243). Hence, SDT might explain how institutional strategies aimed at increasing serious test-taking behavior could work on different levels of motivation.

According to SDT, motivation is conceived of as a continuum ranging from *amotivation* to *extrinsic motivation* to *intrinsic motivation*. In the case of amotivation, the task is perceived as not belonging to the self, and thus no action will be taken. In the state of extrinsic motivation, people do something because they expect a separable outcome. Extrinsic motivation ranges from external regulation to introjection and identification. According to Ryan and Deci (2000), in external regulation, people do something because an external demand is satisfied or they obtain an externally imposed reward contingency. In the state of introjection, people feel a pressure in order to avoid guilt or anxiety or to attain ego enhancements or pride. In the state of identification, people do something because they identify themselves with the personal importance of a behavior and thus accept the regulation as their own. Integration, the next level of SDT, is the state of intrinsic motivation. Intrinsically motivated people do something because it is inherently interesting or enjoyable for them. Intrinsic motivation occurs if the needs for autonomy, competence, and relatedness are satisfied.

In the next paragraphs, we describe those strategies of medical schools to increase serious test-taking behavior that can be explained by SDT. Two strategies that medical schools in Germany and Austria use are consequences and presenting the BPT as an integral part of the medical schools’ assessment. The external regulation level of SDT can explain the assumed effect of both strategies on serious test-taking behavior.

1. Whereas in summative assessment, *consequences* take the form of evaluative test scores, formative assessment focuses on nongrading consequences such as feedback (Black and William 1998) or having the assessment as a prerequisite for enrolling in the following semester’s course (Wise and DeMars 2005) or assessment. In a recent empirical study by Liu et al. (2015), participants in a large-scale assessment assigned to a condition with consequences of their performance for their institution showed more test-taking effort than participants in a control condition. Here, we focus on consequences not related to performance but to the mere participation in the BPT.
2. There are two different ways of how medical schools conducting the BPT present the progress test. Either they present it as part of the *evaluation* or as part of the *assessment*. Evaluation is part of the quality management and has less external regulations than assessment. Presenting the BPT as part of evaluation means: communication about the BPT by the head or coordinator of the quality management/evaluation team and/or supervising the BPT by him/her and/or BPT being part of the evaluation regulations of the medical school. At these schools, the BPT is mainly used as large scale assessment. Presenting the BPT as part of the assessment means: communication about the BPT by the coordinator of assessment and/or supervising the BPT by him/her and/or BPT being part of the examination regulations. Additionally, it can mean that the BPT is introduced in an introductory session of assessment at the beginning of the preclinical and clinical phases of the undergraduate medical training.
3. Some medical schools provide an opportunity for students to discuss BPT results. The discussion of results can range from presenting correct answers in front of the cohort to discussing individual results face to face with a faculty member. A high degree of *individuality of discussed results* is supposed to increase the feeling of pressure in order to avoid guilt (SDT-level of introjection). If students do not take the test seriously, they have to justify their test-taking behavior to, for instance, a mentor or a faculty member. In our experience, most students actually feel guilty if they have to explain why they do not use this opportunity.
4. A similar strategy that was implemented at one school is that students with low performance on BPT have to discuss their results with their *mentor*. There are two explanations using the SDT why this strategy might work. At large universities where students may feel anonymous, a mentoring-system can have an important side-effect, namely that they feel more committed. This is connected to the third component of SDT’s intrinsic motivation: relatedness. On the other hand, students might feel some kind of guilt if they have to explain why they do not perform at their best. This belongs to the introjection component of SDT and is similar to the strategy of individuality of discussed results.
5. Another strategy to increase serious test-taking behavior is to give some kind of *choice*. This can also be explained by SDT: to provide supportive contexts that enhance the feeling of autonomy. Pintrich (2003) suggested using organizational and management structures that encourage personal and social responsibility and provide a safe, comfortable, and predictable environment. Personal responsibility can be encouraged by giving choices, such as whether to take part in the assessment at all or only for a given number of testing times, or by letting students choose between different times or places for the assessment. Voluntary participation as another option of giving choice, however, can lead to low participation rates [e.g., 8% with a range of 0–70% in a semester at one medical school (14 measurement occasions)] and a strong selection bias in the sample and therefore in the averages of the test scores that are used for feedback.

Although all medical schools that conduct the BPT used some of these strategies, the test-taking effort was quite different. Therefore, we sought to identify those institutional factors that were related to test-taking effort to provide medical schools with practical recommendations on how to increase more serious test-taking behavior in low stakes assessments. Because an experimental setting in this field was not applicable, we chose a correlational approach.

The strategies we focused on in this study are:

- (1) Consequences for not participating,
- (2) Presentation type of the BPT (as assessment vs. as evaluation),
- (3) Individuality of discussed results,
- (4) Discussing low test performance with mentor,
- (5) Give choices (e.g. for place or date of test-taking).

## Method

### Sample

The low stakes Berlin Progress Test (BPT) was developed at the Charité Medical School and is available to all 16 participating medical schools in the German and Austrian consortium (Nouns and Georg 2010; Osterberg et al. 2006). All medical schools in this consortium were asked to participate in this study. Nine medical schools agreed.

All participations in the BPT at these nine medical schools from summer semester 2008 to winter semester 2015/2016 (*N* = 31,107, *T *= 16 measurement occasions) were included in this study, resulting in 108,140 observations in total. On average, students participated 3.48 times (*SD* = 2.63) in the BPT, ranging from 1 to 13 times. One faculty member per medical school who was responsible for the administration of the BPT provided data on the employed strategies, which were reconstructed from archives and the memory of the administrators.

### Materials

#### Strategies

We created a questionnaire that contained the strategies described above as selectable options that, which faculty members ticked if the medical school actually had used the respective strategy. Additional activities could be reported in open response fields. We also asked for the timeline if activities had changed over time. Moreover, we asked which semesters were affected if activities varied between semesters, for instance, between the preclinical and clinical phase of undergraduate training. The questionnaire is provided in the Online Supplementary Material.

#### Test-taking behavior

Students who took the BPT were categorized as “serious” (1) or “nonserious” (0) test-takers. Criteria for nonserious test-taking behavior were adopted from Karay et al. (2015): response behavior was classified as nonserious if a student (a) sat the 200-item test in equal to/less than 15 min, or (b) gave no answer at all (i.e., omitted all questions) or, (c) always chose the “don’t know” option, or (d) was identified as a nonserious test-taker by means of a person-fit index. A review of appropriateness measurement showed that the nonparametric group-based index Modified Caution Index (Harnisch and Linn 1981) might be best suited for the use within the BPT data (Brauns 2007). The Modified Caution Index was tested against the Person Conformity Index (PCI), which was especially developed for use with BPT data. In a simulation study, as well as in an empirical study, the Modified Caution Index was tested against the PCI showing that the PCI was superior to the Modified Caution Index according to sensitivity and specificity (Brauns 2007, 2008). The PCI is a group-based index requiring the nonparametric double monotony model and identifies test-takers who answer more frequently questions with higher difficulty than their ability would allow by using a trend test (test of significance) (Brauns 2007, 2008).

### Procedure

We first collected data on the strategies as implemented at the medical institutions from the faculties and then merged this information with students’ data on the test-taking behavior. This procedure is now described in more detail:

In the winter semester 2015/2016, we asked each faculty member responsible for conducting the BPT at all participating medical faculties to fill in our questionnaire on strategies taken to implement the BPT described above. All answers were manually entered into SPSS 24. Answers to open options were categorized into additional strategies we had not anticipated beforehand. The completed data file for each medical school was sent to the corresponding responsible person to correct categorizations if necessary. After correction, the files of all participating schools were merged into one. In the next step, we recoded the answers per strategy into 0 (strategy not used) and 1 (strategy used, see Table 1). The final file from step 1 contained all coded strategies dependent on medical school, time of test taking (e.g., summer 2009), and the semester.

**Table 1:** Operationalization of medical schools’ strategies related to the indicator of taking the low stakes Berlin Progress Test (BPT) seriously

Strategy (with characteristics)	Code
Consequences for not participating	
No consequences	0
No admission to further courses when not taking part	1
No admission to assessment when not taking part	1
Presentation type	
Supervision of BPT on test date by assessment coordinator	1
Communication by assessment coordinator	1
Information about progress testing via email from assessment coordinator	1
Information about progress testing in lecture about assessment	1
Information about BPT being in accordance with examination regulations	1
Communication by head of quality management or by coordinator of quality management/evaluation	0
Supervision of BPT on test date by quality management/evaluation coordinator	0
Information about progress testing via email from quality management/evaluation coordinator	0
Individuality of discussed results	
No individuality	0
Low individuality	1
Moderate individuality	1
High individuality	1
Discussing results with the mentor	
No	0
Yes	1
Give choices	
No choice	0
Choice of date or choice of place	1
Choice of date and choice of place	1

In a second step, the original retrospective data of the BPT from all participating medical schools were pseudo-coded to ensure anonymity. A file was extracted with all students’ pseudo-codes, medical school, time of test taking, semester, and serious/nonserious test-taking classification.

In a third step, the files with student data and the files with data of institutional conditions in the timeline were assembled by medical school, time of test taking, and semester. The resulting file was used for statistical analyses. The Ethical Review Board of the Medical Faculty Mannheim, Heidelberg University, approved the study (2015-833R-MA).

### Analysis

We analyzed relationships between different institutional strategies regarding the implementation of the BPT using a mixed effects logistic regression model within the generalized linear mixed model framework (e.g., Jiang 2007; McCulloch et al. 2008; Stroup 2012) with test-taking behavior being the dichotomous dependent variable (0 = nonserious vs. 1 = serious). The regression model comprises several control variables, whose effects are not of substantive interest, and the variables representing the different institutional strategies as substantively interesting predictors. As control variables, the three study design factors “Medical School”, “Date of Measurement (Wave)”, and “Student” are considered in the analyses. Students are nested within medical schools and repeatedly measured. This is a classic repeated measurement design with one grouping factor (“Medical School”). Thus, in total, there are five sources of variation in the test-taking behavior: “Medical School”, “Students” (within medical schools), “Wave”, “Medical School × Wave”, Residual. In order to control for these sources, all of them were included in our model. The factors “Students” and “Medical School × Wave” were modeled as random effects with an unstructured covariance matrix, whereas “Medical School” and “Wave” were included as deviation coded fixed effects to avoid estimation problems due to their low numbers of units. As substantively interesting predictors, we entered the five dummy-coded institutional strategies as fixed effects into our model: “Consequences for not participating” (0 = no vs. 1 = yes), “Presentation type” (0 = evaluation vs. 1 = assessment), “Individuality of discussed results” (0 = no vs. 1 = yes), “Discussing results with mentor” (0 = no vs. 1 = yes), and “Give choices” (0 = no vs. 1 = yes). Thus, the intercept in our model reflects the predicted logit for an average medical school at an average wave where no consequences for not participating, a presentation of the progress as an evaluation, no individuality of discussed results, no discussion of results with the mentor, and no choices prevail. We additionally considered all two-way interactions^1^ between institutional strategies that were possible to model given the study design and the data, that is, the interaction “Presentation type × Give Choices”. For our model, we carefully checked for multicollinearity among the predictors to avoid unstable parameter estimates and nonconvergence. For discovering multicollinearity, we inspected the variance inflation factor (VIF). All VIF values were below 6, indicating that multicollinearity is not an issue (see, e.g., Kennedy 1992; Kutner et al. 2004; Pan and Jackson 2008).

We estimated our models in the R environment (R Core Team 2019) using the glmer function from the lme4 package (Bates et al. 2015) as well as optimization routines from the nloptr package (Ypma 2017). We selected the binomial family with the logit link, used maximum likelihood estimation based on adaptive Gauss–Hermite quadrature, and chose the bobyqa optimizer from the nloptr package without the calculation of derivatives. We estimated the model with a convergence criterion of 0.000001. Convergence was reached after 4317 iterations.

## Results

### Descriptive statistics

The percentage of nonserious test-takers ranged from 1% (Medical School 2) to 26% (Medical School 9), with the median at 13%. Of all nonserious test-takers 6971 (48%) were identified with the criteria of unanswered questions (b and c), 5327 (37%) with the criterion of time (a) and 2201 (15%) with criterion PCI (d). Numbers of serious and nonserious test-takers per strategy are provided in Table 2.

**Table 2:** Descriptive statistics of strategies

Strategy	*N*_NTTB_ (%)	*N*_STTB_ (%)	*N*_total_
Consequences for not participating			
No	974 (10%)	8817 (90%)	9791
Yes	11,383 (12%)	86,996 (88%)	98,379
Presentation type			
Evaluation	6068 (18%)	27,440 (82%)	33,508
Assessment	5902 (9%)	61,474 (91%)	67,376
Individuality of discussed results			
No	12,069 (14%)	74,938 (86%)	87,007
Yes	288 (1%)	20,875 (99%)	21,163
Discussing results with mentor			
No	12,321 (13%)	85,600 (87%)	97,921
Yes	36 (< 1%)	10,213 (> 99%)	10,249
Give choices			
No	7344 (9%)	73,645 (91%)	80,989
Yes	5013 (18%)	22,168 (82%)	27,181

*NTTB* nonserious test-taking behavior, *STTB* serious test-taking behavior. Note that the reported proportions are of the total sample. Due to potential occurrences of Simpson’s paradoxes, odds and odds ratios from this table might differ, even in sign, from model results reported in Table 3, where results were conditional on medical school as a control variable

### Model

Table 3 shows the results for the estimated model. The regression coefficients of the predictors “Consequences for not participating”, “Discussing results with mentor” and “Give choices” as well as the interaction between “Presentation type” and “Give choices” were all significantly different from 0 (at α = 0.05) while controlling for Wave, Medical School, and Student-specific and Medical School × Wave-specific effects. The predictors “Consequences for not participating”, “Individuality of discussed results”, and “Discussing results with mentor” had *positive* coefficients indicating that a model-implied change in the odds of taking the test seriously is greater than one when a particular strategy is implemented as compared to not having implemented it while controlling for all other variables in the model. Considering the predictor effects conditional on the control variables in detail, the odds of taking the test seriously increases by 153% if there are consequences for not participating in the BPT as compared to no consequences for not participating. When the results of low test performance are discussed with the mentor, the odds of taking the BPT seriously are increased by 1423% as compared to a situation where the results of low test performance are not discussed with the mentor. In contrast, if students are given some choice about modalities of their participation (“Give choice”), their odds of taking the BPT seriously declines by about 1 − exp(− 4.25) = 99% as compared to not providing them with this choice. However, this negative main effect is weakened by the variable “Presentation type”: provided that students are given choice, the odds of taking the BPT seriously when the test was presented as an assessment as compared to being presented as an evaluation, is 11.21 higher than the same odds for students who are not given choice. Inspecting the interaction in more detail and considering only those who are given choice, the odds of taking the BPT seriously increases by 1512% = (exp(2.13 + 0.36 − 4.25 + 2.42)/exp(2.13 − 4.25) − 1) × 100 if the BPT was presented as assessment as compared to if it was presented as evaluation. The main effects for the strategies “Presentation type” and “Individuality of discussed results” had nonsignificant regression coefficients, thus, they are unrelated to the seriousness of the test-taking behavior.

**Table 3:** Model results

Parameter	Est.	*SE*	*P*	exp(Est.)	95% CI	
					LL_exp(Est.)_	UL_exp(Est.)_
Fixed effects						
Intercept	2.13	0.42	< 0.001	8.43	3.73	19.09
Consequences for not participating	0.93	0.46	0.043	2.53	1.03	6.20
Presentation type	0.36	0.37	0.333	1.43	0.69	2.98
Individuality of discussed results	0.81	0.74	0.275	2.24	0.53	9.52
Discussing results with mentor	2.72	0.36	< 0.001	15.23	7.53	30.80
Give choices	− 4.25	0.31	< 0.001	0.01	0.01	0.03
Presentation type × give choice	2.42	0.94	0.010	11.21	1.79	70.04
Random effects						
Person (between person)	2.27					
Medical School × Wave	0.31					
Model fit						
Deviance	59,851.3					
AIC	59,915.3					
BIC	60,222.2					

*AIC* Akaike information criterion, *BIC* Bayesian information criterion, *Est.* estimate. The reported estimate for random effects is the standard deviation. Number of responses = 108,140. Number of persons = 31,107. Fixed effects for the control variables Medical School and Wave are reported in the Online Supplemental Material

## Discussion

Low stakes assessment is becoming more and more important in formative and large-scale assessments. To serve the intended purposes, low stakes assessment needs to deal with the high variation in test-taking effort. Therefore, we investigated strategies that medical schools use to increase serious test-taking behavior in low stakes progress testing. We included only theoretically sound strategies that could be derived from self-determination theory. The strategies that were related to higher odds of taking the test seriously are (in decreasing order): discussion of low performance on BPT with the mentor, consequences for not participating, and give choices of place and date of test taking. Additionally, serious test-taking behavior occurred more if students were given choices *and* if the BPT was presented as assessment or if students were given no choices *and* if the BPT was presented as evaluation.

Including discussing the results of low test performance with the *mentor* could work because talking to a faculty member about the low performance could show that performance is important to someone else and students might want to avoid talking about a lower performance. Depending on whether the student is in the introjection level or intrinsic level of the SDT model, her or his more serious test-taking behavior might be explained by avoiding the feeling of guilt (introjection level) or feeling more related (intrinsic level of motivation).

In accordance with previous research, we found empirical evidence that *consequences* are related to serious test-taking behavior. However, unlike previous research that studied the consequences on performance like grading (Baumert and Demmrich 2001) or consequences of performance for the institution (Liu et al. 2015), to the best of our knowledge, this is the first study showing that even consequences of not participating are also related to serious test-taking behavior in low stakes assessments.

Another strategy that was related to more serious test-taking behavior was giving students the *choice* of place and/or date of taking the BPT. Choices are one of the components of the intrinsic motivation level in the SDT. As a meta-analysis of 41 studies showed (Patall et al. 2008), providing choice can enhance, among others, intrinsic motivation and task performance. In contrast to these prior findings, in our study we showed that if students were given the *choice* of place and time for taking the BPT, they showed more nonserious test-taking behavior. This negative relationship was lessened if the BPT was presented as *assessment* rather than as *evaluation*. The latter finding can be explained if students that showed more serious test-taking behavior in the combination of BPT as assessment at the same time as having no choice about the BPT were on the external regulation level of the SDT. If the BPT is part of the evaluation, students have to be on the intrinsic motivation level to show more serious test-taking behavior. Therefore, the components for the intrinsic motivation level are important here, as is freedom of choice.

The strategy of *individuality of discussed results* was not related to serious test-taking behavior. We assumed the functioning of this strategy by the introjection level of SDT by increasing the feeling of pressure in order to avoid guilt. Although the dialogue with the mentor should have a similar effect on the individuality to discuss results it does not seem to be important in the prediction of test-taking behavior. A possible explanation would be that we have asked faculty members responsible for conducting the BPT whether there was a way to discuss the results of the BPT and then how binding the discussion of these results were. Only the second question was included in the analysis. It may be that there is a proportion of students who do not take advantage of the opportunity to discuss the results and accordingly the individual nature of the results of the discussion cannot influence the test-taking behavior of these students. This is an unknown proportion of unexplained variance and may result in us not being able to consider differences, although there may be differences. It must therefore be ensured that the students who have the opportunity to engage in dialogue do so.

Several *methodical and statistical limitations* need to be acknowledged. First, the study approach had a quasi-experimental character as the medical schools had chosen the strategies to improve progress testing on their own. Thus, causal statements concerning the effects of those strategies on test-taking behavior are improper. Second, the models used in this study were adequate with respect to the research question of whether and to what extent strategies correspond to serious test-taking behavior. However, we did not model trends or effects of strategy changes, primarily because this was not within the research scope of this work but also because the design and the data were too sparse for carving out such effects. Third, generalization of results is limited as medical schools self-selected themselves to participate in the progress test and thus might not be a representative sample of all medical schools. Fourth, there were many more strategies with which medical schools tried to increase the serious test-taking behavior. In our study, we put the focus on strategies whose effect can be linked to the SDT. Fifth, identification of students with nonserious test-taking behavior has its limitation, as person-fit indices do not detect all persons correctly (Li and Olejnik 1997; Meijer 1994, 2003; Meijer et al. 1996; Nering and Meijer 1998; Sijtsma and Meijer 2001). The PCI has proven to be the best fitting person-fit index for BPT data with a specificity of 0.91 and a sensitivity of 0.83 in an empirical study (Brauns 2007). The cut-off for the time criterion was rather strict with an average of 4.5 s reading time per question. We chose those methods to objectively and directly measure nonserious test-taking behavior in contrast to other studies that used self-reporting, with its limitation of socially desirable responses (e.g. Baumert and Demmrich 2001) or indirect measure of performance, with its limitation of correlation with ability (e.g. Heeneman et al. 2017).

Additionally, we do have a limitation of content. In our study we missed the teachers’ perspective. Teachers play an important role in motivating students in low stakes assessment. Several references emphasize the importance of teachers and their role in “determining student motivation” (Kursurkar and ten Cate 2013, p. 904), “the efficacy of any education reform” (Shavelson et al. 2008, p. 310), and the importance of teacher commitment when introducing formative assessment (Gikandi et al. 2011). A strategy to enhance the utility of low stakes assessment might be to commit teachers to the assessment. Teachers can inform students as well as use the results of low stakes assessment in class.

Our findings have *implications* for medical education. We could show that strategies that can be assigned to different levels of motivation in SDT are related to more serious test-taking behavior in a low stakes assessment. Kusurkar et al. (2012) already discussed that motivational theories can be used to design the curriculum more effectively because of more motivated students. In their article they listed motivational theories and provided examples to use these theories for medical education. For example, they suggested recognizing student needs and aligning the curriculum accordingly (Drive theory of Hull; Weiner 1992). In our study we could show that not all strategies belonging to a motivational theory are related to serious test-taking behavior in all conditions. Furthermore, we found evidence that students are on different levels of motivation and therefore motivational strategies can have differentiated effects on different students. Curriculum planners do not only have to provide motivational strategies in medical education including low stakes assessments but also have to take strategies into account that address the different motivational levels from external regulation to intrinsic motivational level. Therefore, we suggest including the motivational stage of students according to self-determination theory in further studies on this subject. There are other motivational theories such as expectancy-value theory (Wigfield and Eccles 2000) that can contribute to finding effective strategies related to test-taking effort in low stakes formative assessment. Measures that are related to those motivational theories might also be worth studying.

As a conclusion, we recommend curriculum planners do the following to increase serious test-taking behavior in low stakes assessments:

- Let low performers discuss their results with a mentor to show that faculty cares.
- Have consequences for not participating to show that the low stakes test is an important part of the curriculum.
- Avoid giving students the choice of place and time for taking the BPT.
- If students must be given the choice of place and time for taking the BPT, then integrate the BPT into the assessment system rather than into the evaluation system.

## Electronic supplementary material

Below is the link to the electronic supplementary material.

Supplementary material 1 (PDF 256 kb)

Supplementary material 2 (DOCX 22 kb)

## Footnotes

1 We also ran five univariable logistic regression models, one for each institutional strategy, in order to check for the potential presence of unmodeled interactions. Results are reported in Table S1 in the Online Supplemental Material. The predictor effects from the univariable models were in the same direction as the corresponding predictor effect from the multivariable model. Thus, there are no unmodeled interactions that would flip the direction (sign) of the main effects in the multivariable model and therefore the direction of all main effects is validly interpretable.
