# Supplementary material for: Institutional strategies related to test-taking behavior in low stakes assessment
Source: Adv Health Sci Educ Theory Pract. 2019 Oct 22;25(2):321–35. doi: 10.1007/s10459-019-09928-y (PMC7210238; doi:10.1007/s10459-019-09928-y)
Supplement: Supplementary file 1 — Supplementary material 1 (PDF 256 kb) [file 10459_2019_9928_MOESM1_ESM.pdf]

Strategies for implementing the Berlin Progress Test in undergraduate medical training

Progress tests are widely used and recognized worldwide. In Germany, Austria and Canada they are low stakes (Wrigley, 2012). Despite the high benefit for the students, the acceptance at the individual locations is very different due to the lack of consequences. This study examines which implementation strategies lead to a high acceptance of the Berlin Progress Test (BPT) from the students' perspective. Subsequently, recommendations for a successful implementation of the BPT will be derived.

Please answer this questionnaire conscientiously. The framework conditions and implementation measures you fill in will then be compared with the acceptance data of the previous BPT. Odds of serious and non-serious test-taking behavior will be calculated.

The raw data of the BPT used will, of course, be archived and evaluated without any identifying number such as matriculation number. Written consent has already been given to use the anonymous data relevant to answer the research question. The results are presented anonymously and in such a way that no conclusions can be drawn about individual sites.

By submitting the completed questionnaire, you agree that the information below may be used for the study "Strategies of successfully implementing low-stakes assessment".

Note on filling out the questionnaire: Double-click on the boxes to activate (tick) or deactivate them. If the answer options do not suffice for the explanation, please use an extra sheet for further information stating the concrete number for which you are providing the information.

1. General information on the site

1.1 For which medical school/medical university are the following details given?

RWTH Aachen University, Faculty of Medicine ☐[1]

University of Duisburg-Essen, Medical Faculty ☐[2]

Medical University of Innsbruck ☐[3]

University of Cologne, Faculty of Medicine ☐[4]

Medical Faculty Mannheim of Heidelberg University ☐[5]

Faculty of Medicine – LMU Munich ☐[6]

Carl von Ossietzky University of Oldenburg, School of Medicine and Health Science ☐[7]

University Hospital Regensburg - Medical Faculty ☐[8]

Witten/Herdecke University, Faculty of Health ☐[9]

Charité – University Medicine Berlin ☐[10]

1.2 Which track do you offer? (if there are several, please fill in several questionnaires)

Traditional track ☐[1]

Reformed (Model) track ☐[2]

Other: ______ ☐[3]

1.3 Number of students

1.3.1 How many medical students are currently enrolled at your location? In the specified track) ______

1.3.2 Has there been a change in the number of students in recent years?
Yes: ☐[1] , No: ☐[0]

1.3.2.1 If yes, when? ______ (year including summer term or winter term)

1.3.2.2 If yes, how many medical students were previously enrolled in the specified track at your location? ______

1.4 Since when do you offer the BPT? ______ (year including summer term or winter term)

1.5 Are there consequences if students do not participate in the BPT?

☐[0] No ☐[1] Yes, namely: ______

2. Integration into the curriculum

2.1 Is the BPT an integral part of the curriculum?

☐[0] No, it is an add-on

☐[1] Yes, it is integrated in the timetable

☐[2] Yes, it is integrated in the timetable and is also displayed as such

2.2 Consistency of question type and teaching

2.2.1 When is the first regular patient contact during the curriculum?

☐[1] 1st semester

☐[2] 2nd semester

☐[3] 3rd semester

☐[4] 4th semester

☐[5] 5th semester

☐[6] 6th semester

☐[7] 7th semester

☐[8] 8th semester

☐[9] 9th semester

☐[10] 10th semester

☐[11] Final year

2.2.2 When does a student at your medical school / medical university regularly take the BPT for the first time?

☐[1] 1st semester

☐[2] 2nd semester

☐[3] 3rd semester

☐[4] 4th semester

☐[5] 5th semester

☐[6] 6th semester

☐[7] 7th semester

☐[8] 8th semester

☐[9] 9th semester

☐[10] 10th semester

☐[11] Final year

2.3 Number of competing assessments

2.3.1 How many assessments does a student have to pass in the pre-clinical phase (or 1st / 2nd academic year or 1st to 4th semester) in the period in which he or she also writes the BPT (+/- four weeks)?

Number: ☐ Multiple-Choice-Tests

Number: ☐ OSCE

Number: ☐ more, namely: ☐

2.3.2 How many assessments does a student have to pass in the clinical phase (or 2nd to 5th academic year or 4th to 10th semester) in the period in which he or she also writes the BPT (+/- four weeks)?

Number: ☐ Multiple-Choice-Tests

Number: ☐ OSCE

Number: ☐ more, namely: ☐

2.4 How often does a student take the BPT once it becomes mandatory?

☐[1] 1x per semester

☐[2] 1x per academic year

☐[3] Other, namely: ☐

3. Introduction of the BPT

3.1 Appreciation by faculty members

3.1.1 Do the faculty members know the BPT?

☐0 No

☐1 Yes, those involved, and that is:

☐2 Yes, namely:

3.1.2 Is the BPT presented in teacher trainings and is its importance explained?

☐0 No

☐1 Yes

3.1.3 Do the faculty members present the BPT as a valuable part of the undergraduate medical training?

☐0 No

☐1 Yes

☐2 I don't know

3.1.4 Are the results of BPT used in research?

☐0 No

☐1 Yes, every now and then

☐2 Yes, regularly/frequently

☐3 I don't know

3.2 Representation by members of higher hierarchical levels

3.2.1 Who communicates with the students about the BPT?

☐1 Dean of Studies

☐2 Head of the Dean's Office of Studies

☐3 Coordinator of Assessment

☐4 Other, namely

3.2.2 Who supervises the BPT on the day of the test?

☐[1] Dean of Studies

☐[2] Head of the Dean’s Office of Studies

☐[3] Coordinator of Assessment

☐[4] Other, research assistants

☐[5] Other, student employees

☐[6] Other, namely ______

3.3 Information about the BPT

3.3.1 How do students receive information about the BPT? (multiple answers possible)

☐[1] Website

On the following pages ______

☐[2] Emails

Sender: ______

☐[3] Postings

Place: ______

☐[4] Through the following central information events

______

☐[5] At the beginning of the test

☐[6] Other, namely ______

3.3.2 What does the information about the BPT contain? (multiple answers possible)

☐[1] Organization

Short key points of the content ______

☐[2] Embedding in the curriculum

Short key points of the content ______

☐[3] Importance / relevance for the students themselves

Short key points of the content

☐[4] Importance / relevance for the institution

Short key points of the content

☐[5] Principle of progress testing

Short key points of the content

☐[6] Other, namely:

Short key points of the content

4. Feedback from the BPT

4.1 Mode of feedback

4.1.1 How do students at your institution get their individual result?

4.1.2 Since when do students at your institution get their individual result in this way?

Since (year including summer term or winter term)

4.1.3 How did the students at your institution get their individual results before?

4.2 Immediate feedback

4.2.1 How is the BPT written at your location?

☐1 Paper-based from to (years including summer term or winter term)

☐2 Computer-based from to (years including summer term or winter term)

4.2.2 Do the students get immediate feedback („Schnellrückmeldung")?

☐0 No

☐1 Yes, since (year including summer term or winter term)

4.2.3 In what form are the most detailed results of the feedback listed?

☐1 Item by item

☐2 Organ- / discipline-based

☐3 Scores (Number of correct answers and test score)

4.3 Dialog option

4.3.1 Is there a way for students to discuss the results?

☐0 No, ☐1Yes, since [ ] (year including summer term or winter term) in the form of:

[ ]

4.3.2 How binding are the results of this discussion?

Strongly binding ☐1☐2☐3☐4☐5☐6☐7☐8☐9☐10 Not binding at all

4.3.3 Is the discussion embedded in other programs, e.g. a mentoring program?

☐0 No, ☐1Yes, since [ ] (year including summer term or winter term) in the form of:

[ ]

5. Other strategies

5.1 What strategies have you used to implement the BPT at your site? And when did you implement them?

**Strategy**	**Semester / Year of Implementation**

5.2 Which strategies do you see as conducive to the acceptance of the BPT?

5.3 Which strategies do you see as hindering the acceptance of the BPT?

Thank you very much for your cooperation!
