# Supplementary material for: Institutional strategies related to test-taking behavior in low stakes assessment
Source: Adv Health Sci Educ Theory Pract. 2019 Oct 22;25(2):321–35. doi: 10.1007/s10459-019-09928-y (PMC7210238; doi:10.1007/s10459-019-09928-y)
Supplement: Supplementary file 2 — Supplementary material 2 (DOCX 22 kb) [file 10459_2019_9928_MOESM2_ESM.docx]

**Online Supplemental Material**

Table S1

*Regression results for univariable regression models versus the full regression model.*

|  | Univariable models | | | | | |  | Multivariable model | | | | | |
| --- | --- | --- | --- | --- | --- | --- | --- | --- | --- | --- | --- | --- | --- |
|  | Est. | *SE* | *p* | exp(Est.) | 95% CI | |  | Est. | *SE* | *p* | exp(Est.) | 95% CI | |
| Parameter |  |  |  |  | LL_exp(b)_ | UL_exp(b)_ |  |  |  |  |  | LL_exp(b)_ | UL_exp(b)_ |
| (Intercept) |  |  |  |  |  |  |  | 2.13 | 0.42 | <.001 | 8.43 | 3.73 | 19.09 |
| Consequences for not participating | 1.730 | 1.00 | 0.083 | 5.64 | 0.80 | 39.84 |  | 0.93 | 0.46 | 0.043 | 2.53 | 1.03 | 6.20 |
| Presentation type | 3.433 | 0.65 | <.001 | 30.98 | 8.72 | 110.08 |  | 0.36 | 0.37 | 0.333 | 1.43 | 0.69 | 2.98 |
| Individuality of discussed results | 3.155 | 0.50 | <.001 | 23.45 | 8.85 | 62.15 |  | 0.81 | 0.74 | 0.275 | 2.24 | 0.53 | 9.52 |
| Discussing results with mentor | 3.121 | 0.47 | <.001 | 22.68 | 9.00 | 57.16 |  | 2.72 | 0.36 | <.001 | 15.23 | 7.53 | 30.80 |
| Give choices | -3.706 | 0.34 | <.001 | 0.02 | 0.01 | 0.05 |  | -4.25 | 0.31 | <.001 | 0.01 | 0.01 | 0.03 |
| Wave s08 |  |  |  |  |  |  |  | 2.96 | 0.35 | <.001 | 19.27 | 9.64 | 38.50 |
| Wave w08 |  |  |  |  |  |  |  | 2.82 | 0.35 | <.001 | 16.71 | 8.45 | 33.07 |
| Wave s09 |  |  |  |  |  |  |  | 2.07 | 0.34 | <.001 | 7.89 | 4.04 | 15.44 |
| Wave w09 |  |  |  |  |  |  |  | 2.60 | 0.34 | <.001 | 13.45 | 6.93 | 26.08 |
| Wave s10 |  |  |  |  |  |  |  | 1.90 | 0.34 | <.001 | 6.66 | 3.40 | 13.03 |
| Wave w10 |  |  |  |  |  |  |  | 2.21 | 0.34 | <.001 | 9.12 | 4.71 | 17.66 |
| Wave s11 |  |  |  |  |  |  |  | 2.48 | 0.35 | <.001 | 11.97 | 6.01 | 23.85 |
| Wave w11 |  |  |  |  |  |  |  | 1.64 | 0.34 | <.001 | 5.16 | 2.65 | 10.02 |
| Wave s12 |  |  |  |  |  |  |  | 1.96 | 0.33 | <.001 | 7.08 | 3.74 | 13.43 |
| Wave w12 |  |  |  |  |  |  |  | 1.46 | 0.32 | <.001 | 4.29 | 2.29 | 8.02 |
| Wave s13 |  |  |  |  |  |  |  | 1.46 | 0.33 | <.001 | 4.31 | 2.28 | 8.15 |
| Wave w13 |  |  |  |  |  |  |  | 1.09 | 0.32 | 0.001 | 2.99 | 1.60 | 5.58 |
| Wave s14 |  |  |  |  |  |  |  | 1.16 | 0.32 | <.001 | 3.18 | 1.68 | 6.01 |
| Wave w14 |  |  |  |  |  |  |  | 1.08 | 0.32 | 0.001 | 2.93 | 1.57 | 5.49 |
| Wave s15 |  |  |  |  |  |  |  | 1.55 | 0.33 | <.001 | 4.72 | 2.48 | 8.97 |
| Medical School 1 | |  |  |  |  |  |  | -2.13 | 0.30 | <.001 | 0.12 | 0.07 | 0.22 |
| Medical School 2 | |  |  |  |  |  |  | -0.97 | 0.55 | 0.080 | 0.38 | 0.13 | 1.12 |
| Medical School 4 | |  |  |  |  |  |  | 2.60 | 0.30 | <.001 | 13.40 | 7.50 | 23.93 |
| Medical School 8 | |  |  |  |  |  |  | -1.91 | 0.40 | <.001 | 0.15 | 0.07 | 0.32 |
| Medical School 9 | |  |  |  |  |  |  | 0.28 | 0.35 | 0.416 | 1.33 | 0.67 | 2.63 |
| Medical School 10 | |  |  |  |  |  |  | -1.16 | 0.32 | <.001 | 0.31 | 0.17 | 0.58 |
| Medical School 11 | |  |  |  |  |  |  | 0.84 | 0.66 | 0.203 | 2.31 | 0.64 | 8.38 |
| Medical School 12 | |  |  |  |  |  |  | 1.21 | 0.37 | 0.001 | 3.34 | 1.60 | 6.95 |
| Presentation type × Give Choice | | | |  |  |  |  | 2.42 | 0.94 | 0.010 | 11.21 | 1.79 | 70.04 |
| Random effects | |  |  |  |  |  |  |  |  |  |  |  |  |
| Person (between person) | |  |  |  |  |  |  | 2.27 |  |  |  |  |  |
| Medical School × Wave | |  |  |  |  |  |  | 0.31 |  |  |  |  |  |
| Model fit |  |  |  |  |  |  |  |  |  |  |  |  |  |
| Deviance |  |  |  |  |  |  |  | 59851.3 |  |  |  |  |  |
| AIC |  |  |  |  |  |  |  | 59915.3 |  |  |  |  |  |
| BIC |  |  |  |  |  |  |  | 60222.2 |  |  |  |  |  |
| Note. AIC = Akaike information criterion; BIC = Bayesian information criterion; Est. = estimate. The reported estimate for random effects is the standard deviation. Number of responses = 108,140. Number of persons = 31,107. | | | | | | | | | | | | | |
